# Ethnicity-based analysis of supragingival plaque composition and dental health behaviours in healthy subjects without caries

**DOI:** 10.1016/j.heliyon.2024.e35238

**Published:** 2024-08-03

**Authors:** Yishan Zhang, Fanghong Liu, Dan Mo, Yiling Jiang, Tian Lin, Sicheng Deng, Jue Lan, Rongmin Qiu

**Affiliations:** College & Hospital of Stomatology, Guangxi Medical University, Guangxi Key Laboratory of Oral and Maxillofacial Rehabilitation and Reconstruction, Guangxi Clinical Research Center for Craniofacial Deformity, Guangxi Key Laboratory of Oral and Maxillofacial Surgery Disease Treatment, Guangxi Health Commission Key Laboratory of Prevention and Treatment for Oral Infectious Diseases, Key Laboratory of Research and Application of Stomatological Equipment, Education Department of Guangxi Zhuang Autonomous Region, No.10 Shuangyong Road, Nanning, Guangxi, 530021, China

**Keywords:** Caries-free, Ethnicity, Plaque microbiota, Dental behaviours, 16S rDNA

## Abstract

**Objective:**

The primary objective of this study was to scrutinise the disparities in the diversity, structure, and function of the oral microbiome among caries-free children from the Zhuang and Han ethnic groups with a focus on the influence of ethnically distinct oral health behaviours on the composition of the oral microbiota.

**Methods:**

A questionnaire survey was conducted to assess oral health behaviours and dental plaque samples were collected from 96 Zhuang and Han children aged 4–5 years living in Guangxi, southern China for high-throughput sequencing. PCR amplification was performed for sequencing of the V4 region of the 16S rDNA gene, and second-generation sequencing was performed using the Illumina MiSeq platform to compare and analyse the diversity, structure and function of the microbiota.

**Results:**

Single-factor analysis revealed significant differences between the Zhuang and Han ethnic groups regarding juice consumption, the frequency of consuming sugar-sweetened food or beverages before bedtime, the age that individuals started toothbrushing, the frequency of toothbrushing and the frequency of parental assistance with toothbrushing (*p* < 0.001). The dominant phyla were *Proteobacteria, Firmicutes*, etc., and the dominant genera included *Streptococcus* and *Neisseria*. The dental plaques of the caries-free Zhuang and Han ethnic groups had similar core microbiomes, with no significant differences in the diversity and structure of the microbiota and no significant differences in the abundance of the dominant genera. In addition, no significant difference in metabolic function was observed between the Zhuang and Han ethnic groups.

**Conclusion:**

The core oral microbiota was consistent in caries-free Zhuang and Han children. Despite differences in dietary habits and oral hygiene behaviours between the Zhuang and Han ethnic groups, with a high frequency of sugary food intake but better oral health behaviours in the Zhuang group, there were no significant differences in the diversity, structure and function of the oral microbiota of caries-free children in the Zhuang and Han ethnic groups.

## 1 Introduction

Healthy humans are colonised by a wide range of commensal organisms that form niche-specific microbiotas [1]. The oral microbiome is home to one of the most complex, dynamic and diverse microbiotas in the human body [2]. The oral cavity harbours different microenvironments, such as tooth surfaces, which are colonised by oral microorganisms collectively referred to as biofilms [3]. Eubiosis and dysbiosis of the oral microbiota are thought to be relevant to an increasing range of health outcomes [4]. There are fewer studies on the correlation between the composition, structure, and function of oral microbes and oral health behaviours in children in a healthy oral state, with most studies focusing on oral microbes in children with caries.

There is preliminary evidence that the oral microbial composition in children varies by ethnicity, dietary intake, and lifestyle [5]. Health differences between ethnicities have long been suggested [6]. To date, limited research has demonstrated the relationship between the oral microbiota and ethnicity. There are notable differences in microbiome diversity among individuals of different ethnicities in the United States, including a greater frequency of bacteria implicated in systemic disorders in certain ethnic groups [7]. Species of the oral microbiota are relatively similar between ethnic groups; however, statistically significant differences in the ratios of bacteria have been observed [8]. Moreover, differences between bacteria are associated with the oral environment in which the colonies are located, and ethnic factors have been shown to positively or negatively affect the structure of the oral microbial community [9,10]. Additionally, the structure of the bacterial microbiota is more susceptible to ethnic factors in caries-free children [11]. Thus, the results differ between studies, suggesting that the effect of ethnic differences on the oral microbiota has not been definitively determined. The distinct microenvironment of the oral barrier in children of various ethnic backgrounds allows individuals to maintain distinctive microbial populations that are controlled by complex signalling systems, as well as host and environmental influences [12,13]. In our previous study, we found that Zhuang children consumed sweet fruit juice more than once a day, always or intermittently consumed desserts and sweet drinks before bedtime, started brushing their teeth before the age of 3, and brushed their teeth more than twice a day; additionally, parents helped their children brush their teeth daily [14]. The detection rate of *Candida albicans* in Zhuang children was greater than that in Han children [15] (40.8 % vs. 29.1 %), which indicated that oral behaviour and dietary factors might affect the oral microbiome in different ethnic groups, but further investigation was warranted. Here, we included ethnicity and oral health behaviour factors to provide insight into interethnic differences in oral health behaviour, as well as differences in oral microbiology. Our study was conducted in the Guangxi Zhuang Autonomous Region, which is a multiethnic autonomous region with predominantly Zhuang and Han ethnic groups, and the ratio of Zhuang to Han individuals was nearly 1:2 [16]. Moreover, this investigation aimed to explore whether core microorganisms are shared between the Zhuang and Han populations. The existence of a “core microbiome” was first proposed in 2007 [17]. In the same year, the US National Institutes of Health (NIH) launched the Human Microbiome Project (HMP), one of the goals of which was to determine whether a core microbiome exists among individuals [18].

In summary, we hypothesised that these children would exhibit a similar core microbiota, that there would be differences in oral microbiota between the Zhuang and Han ethnic groups, and that there would be differences related to ethnic-specific oral hygiene behaviours and dietary factors between the Zhuang and Han ethnic groups to maintain oral microbial ecological balance, that is, a caries-free status.

## 2 Methods

### 2.1 Subject population

This investigation was conducted from Oct. 2017 to Feb. 2018. The caries-free participants were selected from a previous study [19], and they were drawn at random from the three kindergartens in Baize City. The sample size for the statistical study of microbial diversity was calculated with reference to similar studies investigating the diversity of bacterial microbiota in children's plaques [20]; this value was determined based on the mean α-diversity (Shannon's index, etc.), sparse curves, and pre-experimental observations, where k is the number of groups (2), *Z*_1-a/2_ is the fraction of the standard normal distribution of the significance level, with values set as *α* = 0.05, 1-*β* = 0.9, and accounting for the rate of loss of visits, with a final enrolment of 48 children in each ethnic group.

$$n=\frac{{\left(Z_{1-\alpha /2}+Z_{1-\beta /2}\right)}^{2}\sigma^{2}\left(1+1/k\right)}{\delta^{2}}=17$$

The inclusion criterion for all subjects was that all three generations of ancestors were of Zhuang or Han descent. Survey respondents were required to meet the following inclusion criteria.

(i) age 4–5 years;
(ii) residing in the local area for more than 6 months;
(iii) three generations of grandparents that were Zhuang or Han;
(iv) caries-free children;
(v) written informed consent.

exclusion criteria.

(i) had systemic or congenital diseases;
(ii) had an abnormal colour and texture of the oral mucosa or periodontitis;
(iii) had an oral appliance;
(iv) had taken antibiotics within one month before survey;
(v) were unable to cooperate with the investigators.

The study was approved by the Human Ethics Committee of Guangxi Medical University (No. 20220164). All subjects provided written informed consent.

### 2.2 Questionnaire design and data collection

The questionnaire was designed based on the Fourth Chinese National Oral Health Survey Methods [21]. The questionnaire included questions about demographic factors, dietary habits, and oral hygiene behaviours (Supplement 1).

After the questionnaire was designed, 10 eligible subjects were randomly selected for a presurvey, and the final questionnaire was revised after receiving comments from the respondents and experts.

### 2.3 Oral examination

The subjects underwent a clinical oral examination by one professional dentist. The dentist used disposable mouth mirrors attached to an intraoral light and CPI probe. The definition and diagnosis of caries-free patients and DMFT were based on the criteria of the World Health Organisation [22].

The criteria for diagnosing tooth status and coding were as follows.

A. Sound crown. A crown is coded as sound if it shows no evidence of treated or untreated clinical caries.
B. Carious crown. Caries is recorded as present when a lesion in a pit or f i ssure, or on a smooth tooth surface, has an unmistakable cavity, undermined enamel, or a detectably softened floor or wall.
C. Filled crown, with caries. A crown is considered fi lled, with decay, when it has one or more permanent restorations and one or more areas that are decayed.
D. Filled crown, with no caries. A crown is considered filled, without caries, when one or more permanent restorations are present and there is no caries anywhere on the crown
E.Missing tooth, due to caries. This code is used for permanent or primary teeth that have been extracted because of caries and are recorded under coronal status.

### 2.4 Oral microbiome detection

#### 2.4.1 Sample collection

Sampling was performed 2 h after eating in the morning, and the mouth was rinsed with PBS solution. A sterile cotton swab was repeatedly applied twice to the cervical *1/3* of the labial and lingual sides of all primary teeth, immediately placed in 2 ml sterile centrifuge tubes containing 500 μl of PBS solution and placed on ice. Within 4 h of collection, all samples were transported on ice to the laboratory and stored at −80 °C prior to further analysis.

#### 2.4.2 DNA extraction and PCR amplification

Total bacterial genomic DNA was extracted from all samples using the DNeasy PowerWater Kit, the concentration of DNA was determined with the Quant-iT PicoGreen dsDNA Assay Kit, and the quality of DNA extraction was assessed by 0.8 % agarose gel electrophoresis. The primers used were 338F (5-ACTCCTACGGGAGGCAGCA-3) and 806R (5-GGACTACHVGGGTWTCTAAT-3). DNA was examined by 2 % agarose gel electrophoresis and recovered using a gel recovery kit from AXYGEN. The PCR-amplified products were initially quantified by fluorescence via electrophoresis. Based on the fluorescence quantification results, each sample was mixed at the appropriate ratio according to the amount of sequencing required for each sample.

#### 2.4.3 Sequencing library preparation and high-throughput sequencing

Sequencing libraries were prepared using Illumina's TruSeq Nano DNA LT Library Prep Kit. Before sequencing, the libraries were quality-checked using the Agilent High Sensitivity DNA Kit. Quantification was then performed, and the qualified library concentration was at least 2 nM. The qualified gradient dilution of each sequencing library (index sequences were not repeatable) was mixed at the appropriate ratio according to the required sequencing protocol, and the process was carried out on a MiSeq sequencer.

#### 2.4.4 Sequence data processing

The raw downstream data from high-throughput sequencing were first preliminarily screened according to sequence quality; the sequences that passed the initial quality screening were identified and assigned to the corresponding samples based on to primer and barcode information; questionable sequences, such as chimaeras, were removed; and community DNA fragments were subjected to paired-end sequencing using the Illumina MiSeq platform. The raw sequencing data were saved in FASTQ format (the R1.fastq and R2.fastq, Read 1 and Read 2 sequences were paired one by one). The FASTQ-formatted bipartite sequences were first screened for quality one by one using the sliding window method. The window size was 10 bp, the step size was 1 bp, and the sequence was moved from the 5′ end to the first base position. The average quality of bases in the window was required to be ≥ Q20 (i.e., the average sequencing accuracy of bases was ≥99 %). The sequence was truncated from the first window with an average quality value lower than Q20, the length of the truncated sequence was required to be ≥ 150 bp, and no ambiguous base N was allowed (v1.2.7, http://ccb.jhu.edu/software/FLASH/) (Magoc and Salzberg, 2011). We also excluded sequences that 1) had >1 base mismatch in the 5′-end primer or 2) contained the >8 consecutive bases. Subsequently, USEARCH (v5.2.236) was used with QIIME software to verify and eliminate chimeric sequences. The bipartite sequences that passed the quality screening were pairwise concatenated based on overlapping bases. The overlapping bases of both the Read 1 and Read 2 sequences were required to be ≥ 10 bp in length, and no base mismatches were allowed. Finally, based on the index information corresponding to each sample (i.e., the barcode sequence for a small sequence of bases at the beginning of the sequence used to identify the sample), the concatenated sequence was assigned to the corresponding sample (the index sequences are required to match exactly), thus obtaining the effective sequence.

### 2.5 Data analysis

EpiData 3.0 software was used for data collation, and double entry and double checking were used to ensure the accuracy of the data. SPSS 26.0 software was used for statistical analysis. The χ^2^ test was used to compare the oral health behaviour of the Zhuang and Han children. All tests were two-sided, and *p* < 0.05 was considered significant.

Valid sequences from the samples were analysed for clustering and species classification using UPARSE software (V7.0.1001). The obtained sequences were classified into OTUs, and representative sequences from each OTU were used for taxonomic status identification and phylogenetic analysis; the presence of shared taxa in two groups (100 % core threshold) was defined as the core microbiome. The diversity level of each sample was assessed based on the abundance distribution of OTUs in different samples, and the sparse curve was used to reflect whether the sequencing depth met the standard. The specific composition of each sample (group) was analysed at different taxonomic levels (and tested for significant differences between groups); Student's t tests were used to compare the α diversity indices measured by QIIME (Chao1, Shannon indices). Similarity analysis was performed using β-diversity analysis, and bacterial community structure was analysed between groups using UniFrac, visualised by principal coordinate analysis (PCoA). The function adonis(3) in the Vegan package of R v1.1.0 was utilized to analyse the differences in the diversity of microbial communities within samples. The code was as follows:

$$\begin{array}{cc} \text{adonis}(\text{formula}=\text{as}.\text{dist}\left(\text{qiime}.\text{data}\$\text{distmat}\right)\;∼\;\text{qiime}.\text{data}\$\text{map}\left[\left[\text{opts}\$\text{category}\right]\right]\text{,} & \text{permutations}=\text{opts}\$\text{num}\;\text{permutations}) \end{array}$$

Multilevel species discriminant analysis was performed using linear discriminant analysis (LDA) effect size (LEfSe), and differences in microbiome composition between groups were detected using LDA. The LDA cut-off value was 2. The Bonferroni method was used for multiplicity correction. Using Mothur software, Spearman rank correlation coefficients were calculated between dominant genera whose abundance was in the top 80, and correlations were constructed for the relevant dominant genera for which *rho* > 0.6 and *p* value < 0.01; these data were imported into Cytoscape (http://www.cytoscape.org/) software for visualisation. Microbial functions were predicted using PICRUSt and compared with the Kyoto Encyclopedia of Genes and Genomes (KEGG) database.

## 3 Results

### 3.1 Comparison of oral health behaviours

A total of 96 caries-free children were included in this study (male: female = 1:1, Zhuang: Han = 1:1). A comparison of oral health behaviours between caries-free Zhuang and Han children aged 4–5 years is shown in Table 1. Caries-free Zhuang children consumed significantly more juice, consumed significantly more sugar-sweetened food or beverages before bedtime, started toothbrushing at a significantly younger age, and exhibited significantly greater frequencies of toothbrushing and parental assistance with toothbrushing than caries-free Han children (*p* < 0.001). A total of 60.4 % of Zhuang parents helped their children brush their teeth every day, compared to only 20.8 % of Han parents (*p* < 0.001). There was no statistically significant difference in the effectiveness of toothbrushing between Zhuang and Han children in terms of the intake of sweets, fresh fruits and vegetables, or carbonated beverages such as coke or in parental checks on the effectiveness of children's toothbrushing (*p*＞0.05).

**Table 1:** Comparison of the oral health behaviours of Zhuang and Han children.

Variable	Zhuang N(%)	Han N(%)	*x*^2^*value*	*p value*
Gender				
Male	24(25.0)	24(25.0)		
Female	24(25.0)	24(25.0)	0.00	1.00
Age				
4 years old	24(25.0)	24(25.0)		
5 years old	24(25.0)	24(25.0)	0.00	1.00
Frequency of fruit, vegetable intake				
＜1 time per day	3(6.2)	4(8.3)		
≥1 time per day	45(93.8)	44(91.7)	0.154	0.695^a^
Frequency of juice intake				
＜1 time per day	30(62.5)	47(97.9)		
≥1 time per day	18(37.5)	1(2.1)	18.964	**＜0.001**^b^
Frequency of carbonated beverage intake				
＜1 time per day	46(95.8)	48(100)		
≥1 time per day	2(4.2)	0(0.0)	2.043	0.247^b^
Frequency of sugar-sweetened food or beverages before bedtime				
Never	16(33.3)	38(79.2)		
Often/Occasionally	32(66.7)	10(20.8)	20.487	**＜0.001**^a^
Age to start toothbrushing				
＜3years	28(58.3)	10(20.8)		
≥3years or not yet	20(41.7)	38(79.2)	14.113	**＜0.001**^a^
Frequency of toothbrushing				
≥2 times per day	32(66.7)	6(12.5)		
≤1 time per day	16(33.3)	42(87.5)	29.445	**＜0.001**^a^
Frequency of parental assistance with toothbrushing				
Everyday	29(60.4)	10(20.8)		
Weekly, sometimes, never	19(39.6)	38(79.2)	15.590	**＜0.001**^a^
Check the effectiveness of children toothbrushing				
Everyday	11(22.9)	7(14.6)		
Weekly, sometimes, never	37(77.1)	41(85.4)	1.094	0.296^a^

a By chi-square test.

b By Fisher's exact test.

### 3.2 Comparison of the oral microbiome

#### 3.2.1 Taxonomic analysis

**Fig. 1:**
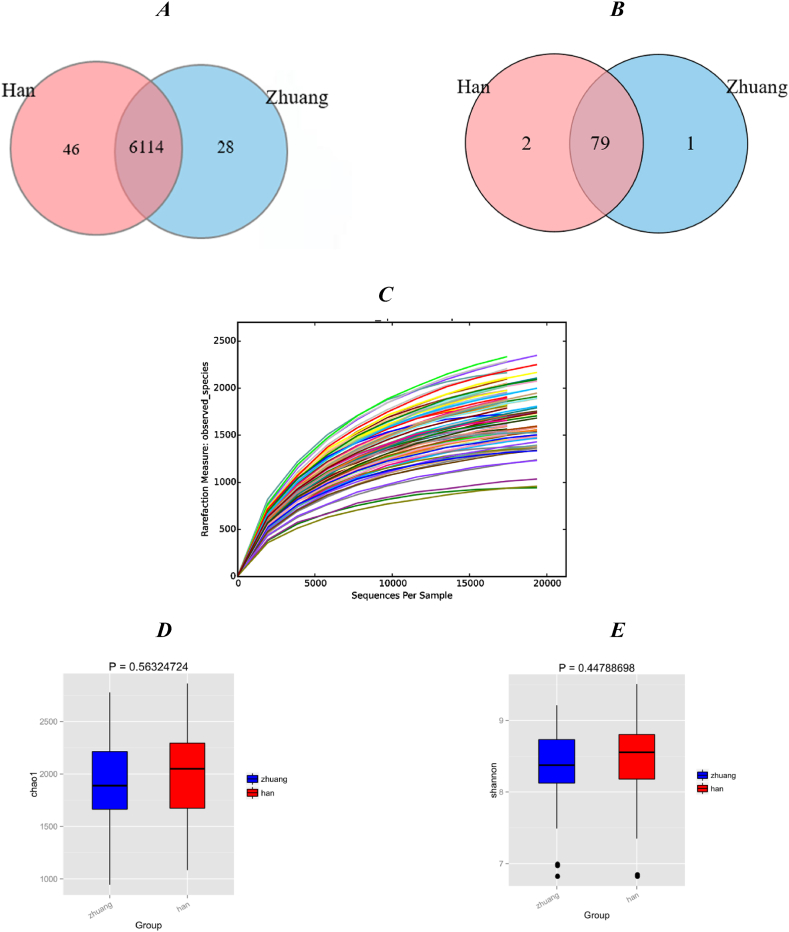
| Venn diagram of the species level (*A*). Venn diagram of the genus level (*B*). Rarefaction curve *(C).* Boxplots of alpha diversity indices reflect bacterial abundance and evenness. Chao1 and Shannon indices are shown for each comparison. Boxplots depict the median and upper and lower quartiles of the samples grouped by Zhuang and Han individuals *(D-E).*

After data trimming and quality filtering, 3658443 valid sequences were generated from 96 supragingival dental plaque samples, with an average of 37796 sequences per sample. The average sequence length was 315.9 bp (Supplement 2). All high-quality sequences with 97 % identity were clustered. A Venn diagram of the species composition revealed 6142 OTUs in the Zhuang ethnic group and 6160 OTUs in the Han ethnic group. A total of 6114 OTUs were shared between the groups, accounting for more than 99 % of all OTUs (Fig. 1 A). Venn diagram of the genus level (Fig. 1 B). The rarefaction curve (Fig. 1C) showed that the number of new OTUs increased with increasing sequencing volume, which indicated that the current sequencing depth was sufficient to capture the bacterial diversity of dental plaques.

#### 3.2.2 Alpha diversity analysis

The alpha diversity indices Chao1 and Shannon's evenness are shown in Table 2. There was no significant difference in the abundance or diversity of oral plaque between the two groups according to the Chao1 (*p* = 0.56), Shannon (*p* = 0.48) indices (Fig. 1D–E).

**Table 2:** Alpha diversity indices of Zhuang and Han.

Diversity Indices	Chao1	Shannon	*P-value*
Zhuang	1955.15 ± 1009.08	8.17 ± 1.34	0.56
Han	1796.64 ± 714.10	7.96 ± 1.14	0.45

#### 3.2.3 Beta diversity analysis

**Fig. 2:**
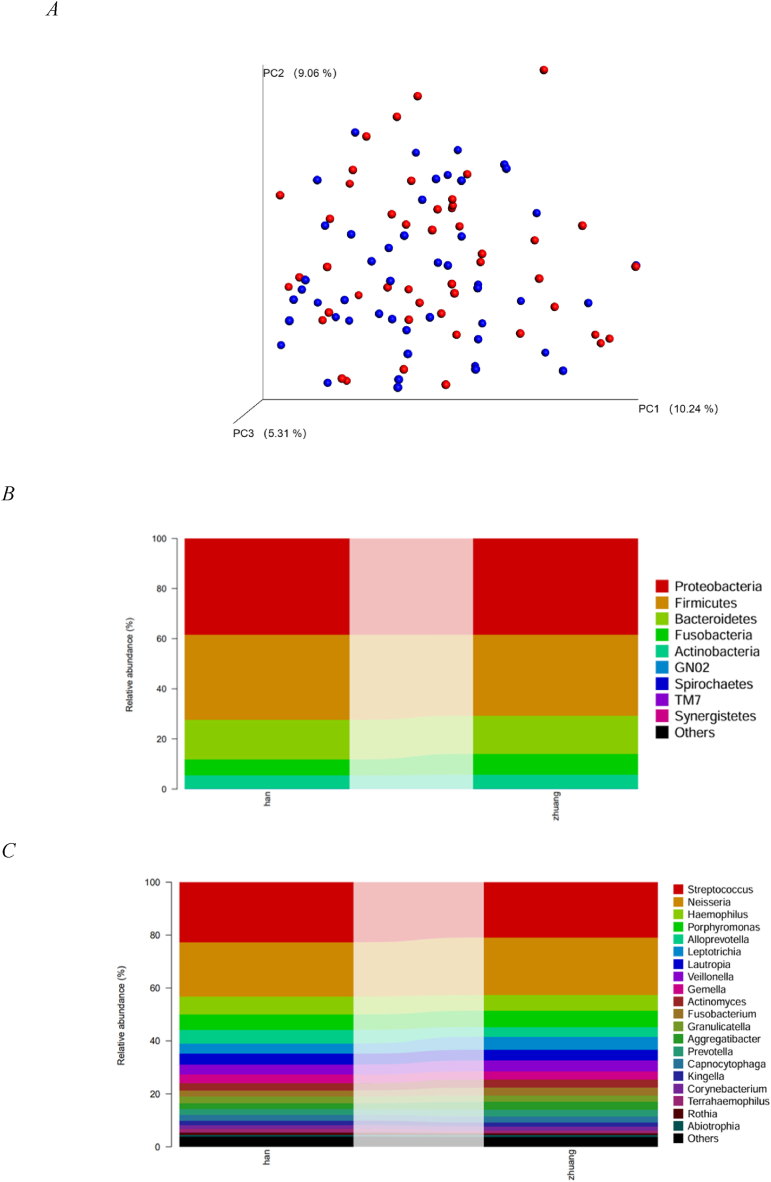
| Principal coordinate analysis of unweighted Euclidean distances between the samples based on relative abundances of OTUs. The closer the distance between two points is, the more similar and less different the microbial community structure between the two samples. Sample data taken from individual studies show clear clustering. Distributions of the predominant bacteria. The results at the phylum level (B). The results at the genus level (C). The predominant taxa (relative abundance >1 % on average) are shown.

To gain insights into similarities in bacterial community structures between the Zhuang and Han ethnic groups, PCoA was performed based on unweighted UniFrac distances. The results showed no clear segregation in community structures between the two groups (Fig. 2), and the percentages in brackets on the coordinate axes represent the proportion of variation in the raw data that can be explained by the corresponding principal components. Based on the PCoA results (Fig. 2 A), no statistically significant differences were detected in the structure of the supragingival plaque microbial community between the Zhuang and Han ethnic groups (*p* > 0.05).

#### 3.2.4 Bacterial community structure

Fig. 2 shows the taxonomic distributions of the predominant bacteria (those with a relative abundance >1 % of the total sequences) at different taxonomic levels based on OTUs. At the phylum level (Fig. 2 B), the 5 most dominant phyla in the Zhuang and Han ethnic groups were Proteobacteria (38.41 % vs. 38.41 %), Firmicutes (33.94 % vs. 32.31 %), Bacteroidetes (15.87 % vs. 15.29 %), and Fusobacteria (8.26 % vs. 6.36 %), accounting for more than 99 % of all sequences. The remaining phyla were detected in a few samples at low rates, with no statistically significant differences between samples (*p* > 0.05). At the genus level (Fig. 2C), 82 genera were detected, of which 18 exhibited an abundance >1 %, in the order of *Streptococcus*, *Neisseria*, *Haemophilus*, *Porphyromonas*, *Alloprevotella*, *Leptotrichia*, *Lautropia*, *Veillonella*, *Gemella*, *Actinomyces*, *Fusobacterium*, *Granulicatella*, *Aggregatibacter*, *Prevotella*, *Capnocytophaga*, *Kingella*, *Corynebacterium*, and *Terrahaemophilus*. *Streptococcus*, *Neisseria*, *Haemophilus*, and *Plasmodium* were present at greater than 5 % abundance. Nevertheless, *Streptococcus* spp. (22.7 % vs. 20.9 %, Han vs. Zhuang) and *Neisseria* spp. (20.5 % vs. 21.7 %, Han vs. Zhuang) exhibited greater than 20 % abundances in each group, suggesting that they may be resident genera associated with oral health.

**Fig. 3:**
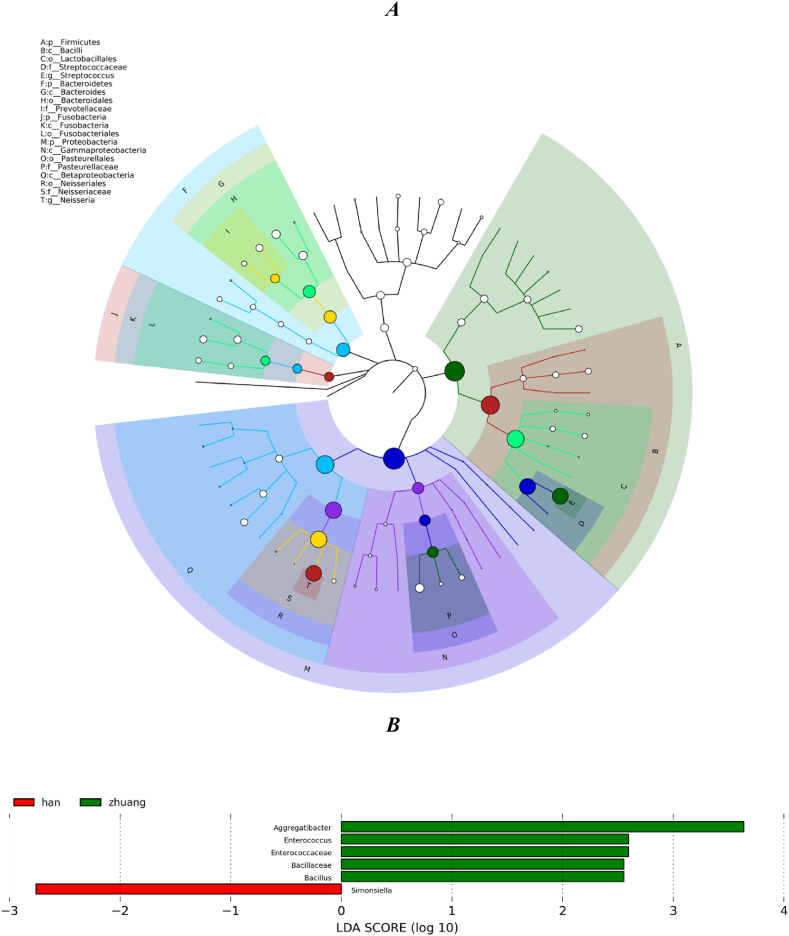
| Tree showing the hierarchical relationships of all taxonomic units from phylum to genus, with the top 20 taxonomic units in terms of relative abundance (A). LEfSe analysis of Han and Zhuang populations from the phylum to genus level (B).

The coloured nodes in Fig. 3 represent the top 20 taxonomic units in terms of relative abundance, including *Streptococcus mutans*, and the size of the nodes in the tree corresponds to the average relative abundance of that taxonomic unit. The distributions of predominant bacteria in both the Han and Zhuang ethnic groups were essentially identical, but notable differences in their relative abundances were observed. To identify genera that were differentially abundant in each group as potential biomarkers, we employed the LEfSe method, using a logarithmic LDA score of 2.0 as the threshold for discriminative features. At the genus level, *Simonsiella* was found to be significantly more abundant in the Han group, while *Aggregatibacter*, *Enterococcus*, and *Bacillus* were more abundant in the Zhuang group. At the family level, *Enterococcaceae* and *Bacillaceae* were significantly more abundant in the Han group (LDA >2, *p* < 0.05; Fig. 3).

#### 3.2.5 Co-occurrence network analysis and functional predictions

**Fig. 4:**
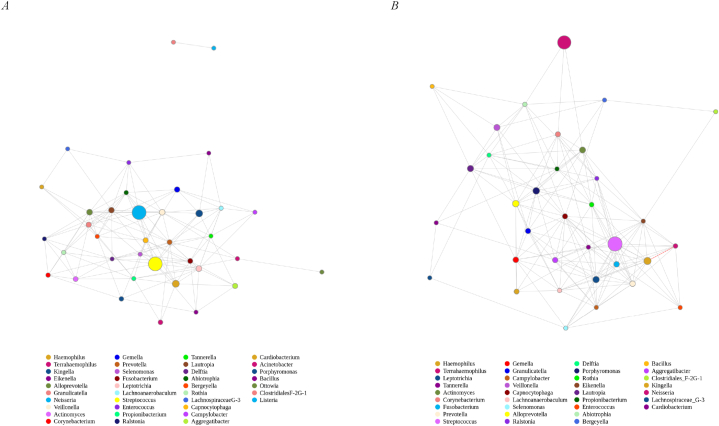
| Network analysis showing the interactions between genera (|SpearmanCoef| > 0.6 and *p* < 0.05). The red line represents strong positive correlation, the green line represents strong negative correlation, and the grey line represents weak correlation. Network analysis of the Zhuang individuals (A). Network analysis of the Han individuals (B).

We conducted a co-occurrence network analysis to determine the relationships among the plaque microbiota at the genus level (Fig. 4A–B). Each point represents a feature, the size of the point is positively correlated with the relative abundance, and the different colours represent different annotations; the red connecting line represents a strong positive correlation, the grey connecting line a strong negative correlation, and grey represents a weak correlation between abundant genera (*p* < 0.05). Among Han children, *Haemophilus* and *Terrahaemophilus* were strongly positively correlated, while other genera were weakly correlated.

**Fig. 5:**
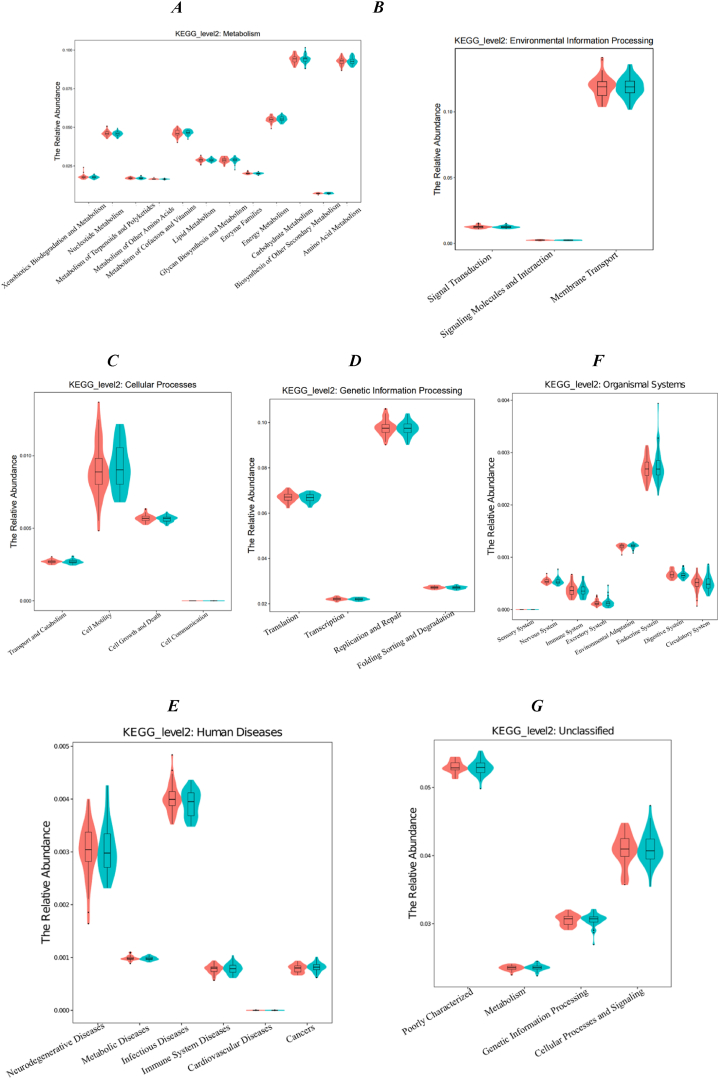

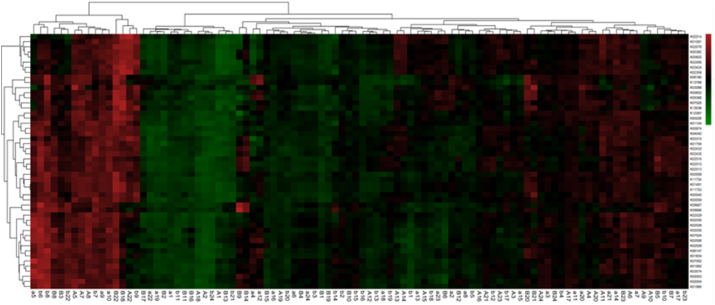
| Bacterial function prediction by PICRUSt analysis (*A-G*, green-Zhuang, red-Han). Bacterial function prediction by PICRUSt analysis and heatmap *(H*).

To predict the functions of the bacteria in the supragingival plaque community, we employed PICRUSt analysis using the 16S rDNA composition data of each sample. The functional predictions derived from the Zhuang-Han comparison did not reveal significant differences (*p*＞0.05) (Fig. 5A–G). The similarity of multiple samples in terms of functional gene composition is reflected by the colour gradient and the degree of similarity (Fig. 5H).

## 4 Discussion

In this study, we analysed the oral microbiota composition of caries-free children from the Zhuang and Han ethnic groups residing in Guangxi. The Venn diagram illustrates the presence of a core microbiota that is shared between Zhuang and Han children, despite the vast diversity of bacteria observed. The appropriate definition of the core microbiome depends on the ecological question addressed [23]. Therefore, in this study, we typically defined the core microbiome as the group of members shared among microbial communities. The predominant phyla observed in the oral microbiota were *Proteobacteria*, *Firmicutes*, *Bacteroidetes*, and *Fusobacteria*, and these core microbiota each accounted for 99 % of the sequences detected between the two groups, which is similar to the results of previous studies [[24], [25], [26]]. The core microbiota of the Zhuang and Han ethnic groups greatly overlapped, indicating that the core oral microbes were in a steady state across the different ethnicities. Several studies have demonstrated that a stable core microbiota is maintained in the oral cavity of healthy individuals for several years [27,28]. The core microorganisms of a healthy oral environment play a role in resiliency in the face of external factors that may cause imbalances in the microbiota [29]. The major difference between healthy individuals and those with caries is in the acid-tolerant population that is selected for during disease progression rather than the predominant microbiota that includes the core microbiome [30]. Thus, individuals in the healthy oral state exhibit a steady core microbiota.

The alpha diversity indices showed that the observed species were not significantly different between the groups. However, there was some significant difference in alpha diversity between children with caries and healthy individuals [31]. The composition of the microbiome of individuals with caries indicates a tendency towards lower α diversity and richness [32], and community diversity in the plaque of healthy individuals was greater than that in individuals with oral diseases such as dental caries. However, whether the low community diversity is a consequence or cause of oral disease within this group remains unclear [33,34]. In the present study, the PCoA results showed no significant differences in beta diversity between the Zhuang and Han ethnic groups, which suggested conservation of the oral microbiota. Meta-analysis revealed significant differences in the beta diversity of bacteria in individuals with caries compared to that in healthy individuals [31]. There was no significant difference in the structure and diversity of the ecological microbiota between the Zhuang and Han populations. We hypothesise that under caries-free conditions, self-classification of ethnicity is a complex paradigm that encompasses many facets; as such, we propose that the diversity of bacterial communities and structures in individuals from different ethnicities might present similarities [10].

*Streptococcus* and *Neisseria* had the greatest proportions between the two populations, which may suggest that they are some of the bacteria capable of preserving a balanced oral microbiome in the Zhuang and Han ethnic groups [35]. For example, *Streptococcus* was found to be the dominant genus in healthy Chinese children [36]. In a study of carious and caries-free children under 6 years old, differential abundance analyses showed that samples from caries-free children were enriched with *Neisseria* [35]. In addition, we found that *Streptococcus* had a lower abundance in the Zhuang group than in the Han group, although this finding did not indicate a difference in the likelihood of dental caries between the Han and Zhuang ethnic groups. This result confirms that ethnic disparities influence the abundance of members of the bacterial microbiota.

Previous studies have shown separate clustering of Korean and Japanese individuals, who also exhibited notable differences in diet and environmental factors [37]. In this study, we found that compared with Han children, Zhuang children consumed juices at a greater frequency and sugar-sweetened foods or beverages before bedtime. Regular sugar intake increases the abundance of acid-producing bacteria and acid-resistant bacteria, which affects colonisation of the normal oral microbiota [38] and ultimately leading to relative differences in the abundance of bacteria in the oral microbiota between Zhuang and Han children. In conclusion, ethnic dietary differences play a role in determining the effects of relative differences in the abundance of the oral plaque microbiota in caries-free children. A previous study suggested that the frequency of sugary drink intake by 1- to 6-year-old children was significantly related to the variation in the dental plaque microbiota in the children [39]. This comparison demonstrated that Zhuang children brush their teeth more thoroughly than Han children and that Zhuang parents are more diligent in assisting their children with toothbrushing. Toothbrushing, a positive health behaviour, is the primary method of mechanical plaque removal. In general, a study noted that the abundance of the oral microbiota was affected by toothbrushing once or twice daily [40]. Oral health behaviours such as dietary habits and oral hygiene behaviours can play an important role in influencing changes in the structure of the oral microbial community [41]. A detailed correlation analysis between the oral microbiota, diet and oral hygiene behaviours is thus warranted. The frequent consumption of sweet foods affects biofilm adhesion and the duration that bacteria are active. Additionally, these foods impact the biofilm microbiota and are related to the functional role of the dominant microbiota. In addition, children with caries had greater utilisation of carbohydrates than did those without caries, and the difference in carbohydrates between the Zhuang and Han populations was not statistically significant, although the frequency of sweet food intake in the Zhuang population was greater than that in the Han population [42]. Nevertheless, the PICRUSt results showed no significant differences between the two populations, so we hypothesise that oral health behaviours play a role in hindering or preventing the function of the microbiota. The combination of dietary habits and oral health behaviours resulted in the microbiota diversity and structure that did not differ significantly between the Zhuang and Han ethnic groups. Furthermore, the PICRUSt results showed that there were no significant differences between the Zhuang and Han ethnic groups in functional pathways such as the metabolism of cofactors and vitamins, sugar biosynthesis and metabolism, and lipid metabolism, which may be influenced by the widespread core microbiome [43].Different levels of 13 functional genes, such as those involved in lipid metabolism and cofactor biosynthesis, are present in healthy individuals and may be involved in maintaining health [44]. Accumulating evidence has indicated that differences in the relative abundance of metabolic pathways can indicate health or specific disease states [45].

Although the dominant genera were similar and the diversity of microbiomes did not differ significantly, there were significant differences in abundance between some nondominant microbiota in the Zhuang and Han populations. The potential biomarkers of the different groups were identified by LEfSe. The results demonstrated that the relative abundance of *Simonsiella*, which was the key biomarker in the Han population, was greater in the Han population. *Simonsiella*-enriched populations have significantly greater dietary intakes of fat and protein, but not sugar. This trend corresponds to a lower frequency of carbohydrate intake in Han children than in Zhuang children [46]. Another study of caries-free children showed that *Simonsiella* was overrepresented in caries-free subjects [47]. The relative abundances of *Bacillus*, *Enterococcus*, and *Aggregatibacter*, which were key biomarkers in the Zhuang population, were greater in the oral microbiome of the Zhuang ethnic group. *Aggregatibacter* is associated with dental caries [48] and can be detected in dental plaque. Although these microbes were detected at low frequencies, this finding highlights an important possibility: even low-abundance members may still act as crucial genera in the behaviour of complex communities [8]. At the family level, *Bacillaceae* and *Enterococcaceae* were more abundant in the Zhuang population. The results of this study demonstrated that the influx of oral strains from phylogenetically diverse microbial taxa into the gut microbiome is extensive in healthy individuals, exhibiting a high degree of variation between subjects [49]. *Enterococcaceae* are generally associated with gut diseases and were significantly more abundant in the Zhuang ethnic group than in the Han ethnic group. Therefore, we should more closely scrutinise the relationships among the oral microbiota, the gut microbiota and the effects of oral hygiene behaviours among caries-free Zhuang and Han children in the future.

## 5 Conclusion

This study provides a thorough analysis of bacterial diversity and community structure between the Zhuang and Han ethnic groups based on plaque samples collected from caries-free children. We found that the "core microbiota" existed between the dental plaques of Zhuang and Han children. Notably, the dental plaque microbiota of caries-free Zhuang and Han children did not differ significantly in terms of diversity, structure and function. These findings suggest that these oral bacteria protect and maintain the balance of the oral microbiota. Further exploration revealed significant differences in oral health behaviours. Thus, our findings suggest that a combination of carbohydrate-rich dietary factors and good oral hygiene promotes the maintenance of a balanced oral microbiota. This study is expected to provide new approaches for preventive and therapeutic strategies. However, avoiding the influence of potential PCR bias and gene profile variation among related genomes remains difficult.

## Limitations

We collected supragingival plaque samples in this study. The collection of plaque samples for 16S rDNA gene analysis can be challenging due to potential PCR bias and gene profile variation among related genomes [50]. This bias can be influenced by factors such as the annealing temperature and number of PCR cycles, as well as interference from DNA flanking the template region [51]. However, the results of the present study revealed no significant differences in the structure and function of the supragingival plaque community among the different populations. Future studies with larger cohorts will be performed to confirm our findings. In addition, changes in the oral plaque microbiota with age need to be validated and explored over an extended period. The interactions between the plaque environment and microorganisms need to be systematically investigated.

## Funding

This work was supported by a grant from the 10.13039/501100011827Guangxi Medical University Science Fund for Young Scholars (GXMUYSF: No. 201320).

## Ethics approval and consent to participate

The study design and protocol were approved by the Ethics Committee of Guangxi Medical University, and written informed consent for participation was obtained from all participants and from their legal guardians.

## Data availability statement

The labelled dataset used to support the findings of this study is available from the corresponding author upon request.

## CRediT authorship contribution statement

**Yishan Zhang:** Writing – original draft, Software, Methodology, Investigation, Formal analysis, Data curation, Conceptualization. **Fanghong Liu:** Writing – original draft, Resources, Project administration, Methodology, Investigation. **Dan Mo:** Investigation, Conceptualization. **Yiling Jiang:** Visualization, Validation. **Tian Lin:** Supervision, Resources. **Sicheng Deng:** Visualization, Resources. **Jue Lan:** Project administration, Investigation. **Rongmin Qiu:** Writing – review & editing, Methodology, Funding acquisition, Conceptualization.

## Declaration of competing interest

We affirm that no author has a financial affiliation or involvement with any commercial organization with a direct financial interest in the subject or materials discussed in this manuscript. Any other potential conflict of interest is disclosed.

## Footnotes

Appendix A Supplementary data to this article can be found online at https://doi.org/10.1016/j.heliyon.2024.e35238.
