# Supplementary material for: Ethnicity-based analysis of supragingival plaque composition and dental health behaviours in healthy subjects without caries
Source: Heliyon. 2024 Aug 3;10(15):e35238. doi: 10.1016/j.heliyon.2024.e35238 (PMC11336456; doi:10.1016/j.heliyon.2024.e35238)
Supplement: Supplementary file 3 [file mmc3.docx]

Questionnaire on oral health behaviours of 4-5 years old children in Baise

Name Sex Ethnicity

Birthday

These are some questions about your child and your oral health behaviours:

1. What is your child's usual frequency of eating the following foods or beverages?

|  | ≥2 times per day | 1 time per day | 2-6 times per week | 1 time per week | 1-3 times per month | never |
| --- | --- | --- | --- | --- | --- | --- |
| Frequency of fruit, vegetable intake |  |  |  |  |  |  |
| Frequency of juice intake |  |  |  |  |  |  |
| Frequency of carbonated beverage  intake |  |  |  |  |  |  |

2. What is your child's frequency of sugar-sweetened food or beverage consumption?

1) □ Often 2) □ Occasionally 3) □ Never

3. When your child start toothbrushing?

1）□ Not yet 2）□ ＜3 years old 3) □ ≥3 years old

4. What is your child's frequency of toothbrushing?

1)□ < 1 time per day 2) □ 1 time per day 3) □ ≥2 times per day

5. Have you taken any of the following steps for brushing your child's teeth?

1 2 3 4

Everyday Weekly Sometimes Never

1）Assistance in children toothbrushing □ □ □ □

2）Check the effectiveness of children toothbrushing □ □ □ □

Please answer the following questions to provide basic information about yourself and your family.

1. The ethnicity of the child is:

1) □ Zhuang 2) □ Han 3) □ Other ethnicities

2. The ethnicity of the father is:

1) □ Zhuang 2) □ Han 3) □ Other ethnicities

3. The ethnicity of the mother is:

1) □ Zhuang 2) □ Han 3) □ Other ethnicities

4. The ethnicity of the child's grandfather is:

1) □ Zhuang 2) □ Han 3) □ Other ethnicities

5. The ethnicity of the child's grandmother is:

1) □ Zhuang 2) □ Han 3) □ Other ethnicities

6. The ethnicity of the child's maternal grandfather is:

1) □ Zhuang 2) □ Han 3) □ Other ethnicities

7. The ethnicity of the child's maternal grandmother is:

1) □ Zhuang 2) □ Han 3) □ Other ethnicities
