# Supplementary figures and images for: Ethnicity-based analysis of supragingival plaque composition and dental health behaviours in healthy subjects without caries
Source: Heliyon. 2024 Aug 3;10(15):e35238. doi: 10.1016/j.heliyon.2024.e35238 (PMC11336456; doi:10.1016/j.heliyon.2024.e35238)

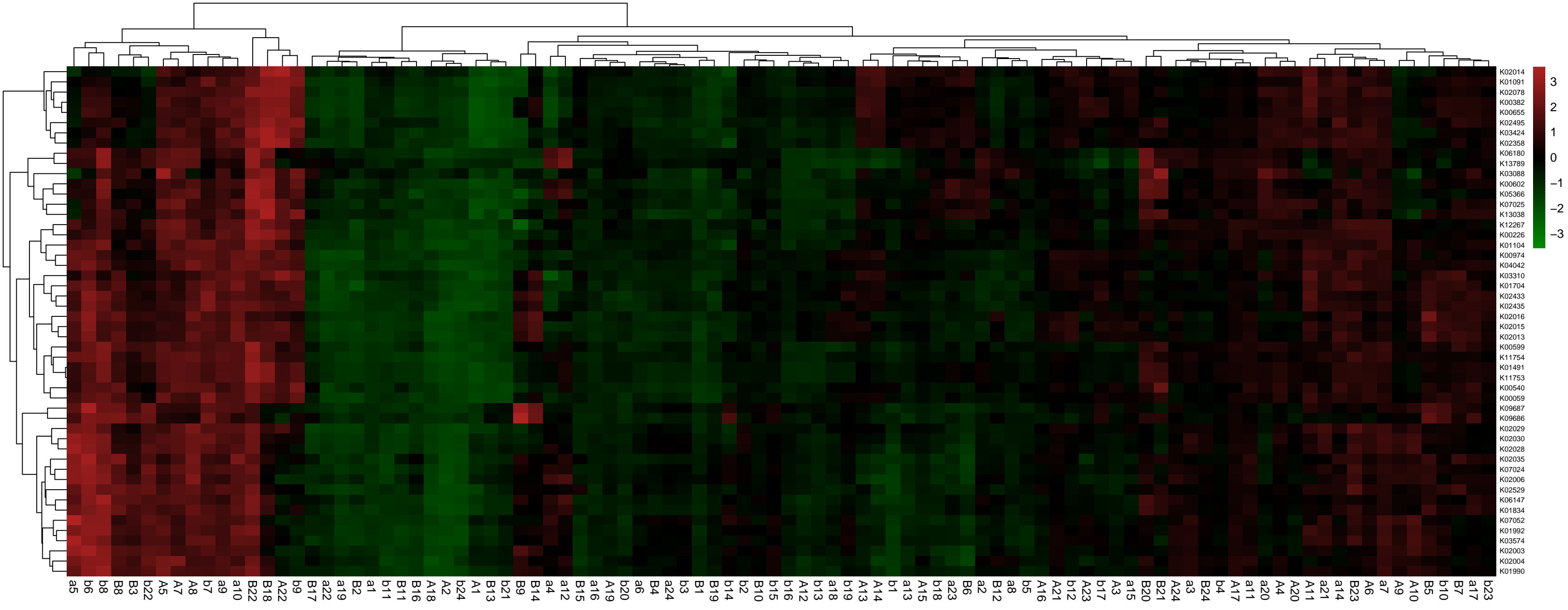

Supplement: Supplementary file 5 [file mmc5.pdf]

# KEGG\_level2: Cellular Processes

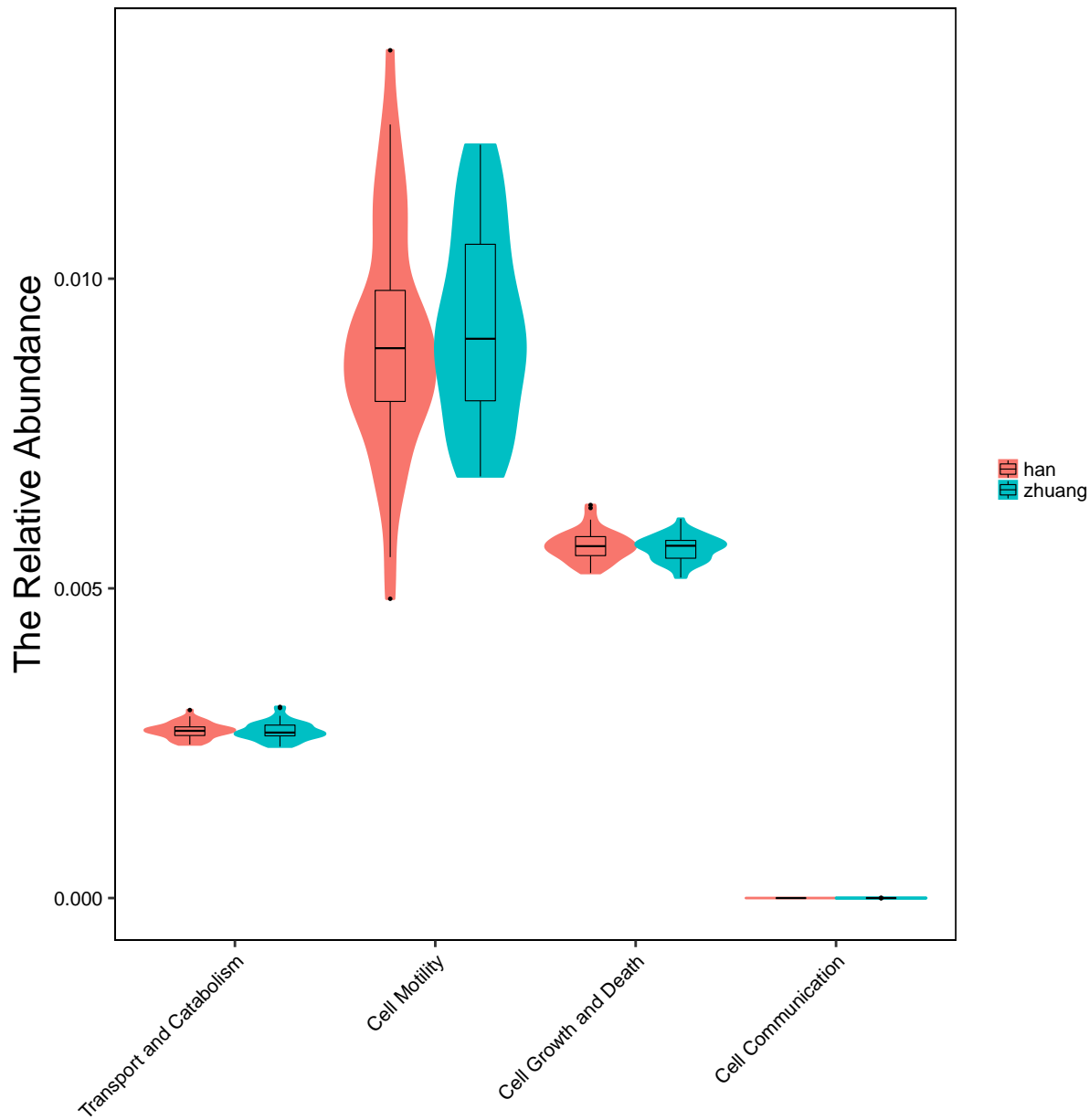

Supplement: Supplementary file 6 [file mmc6.pdf]

# KEGG\_level2: Environmental Information Processing

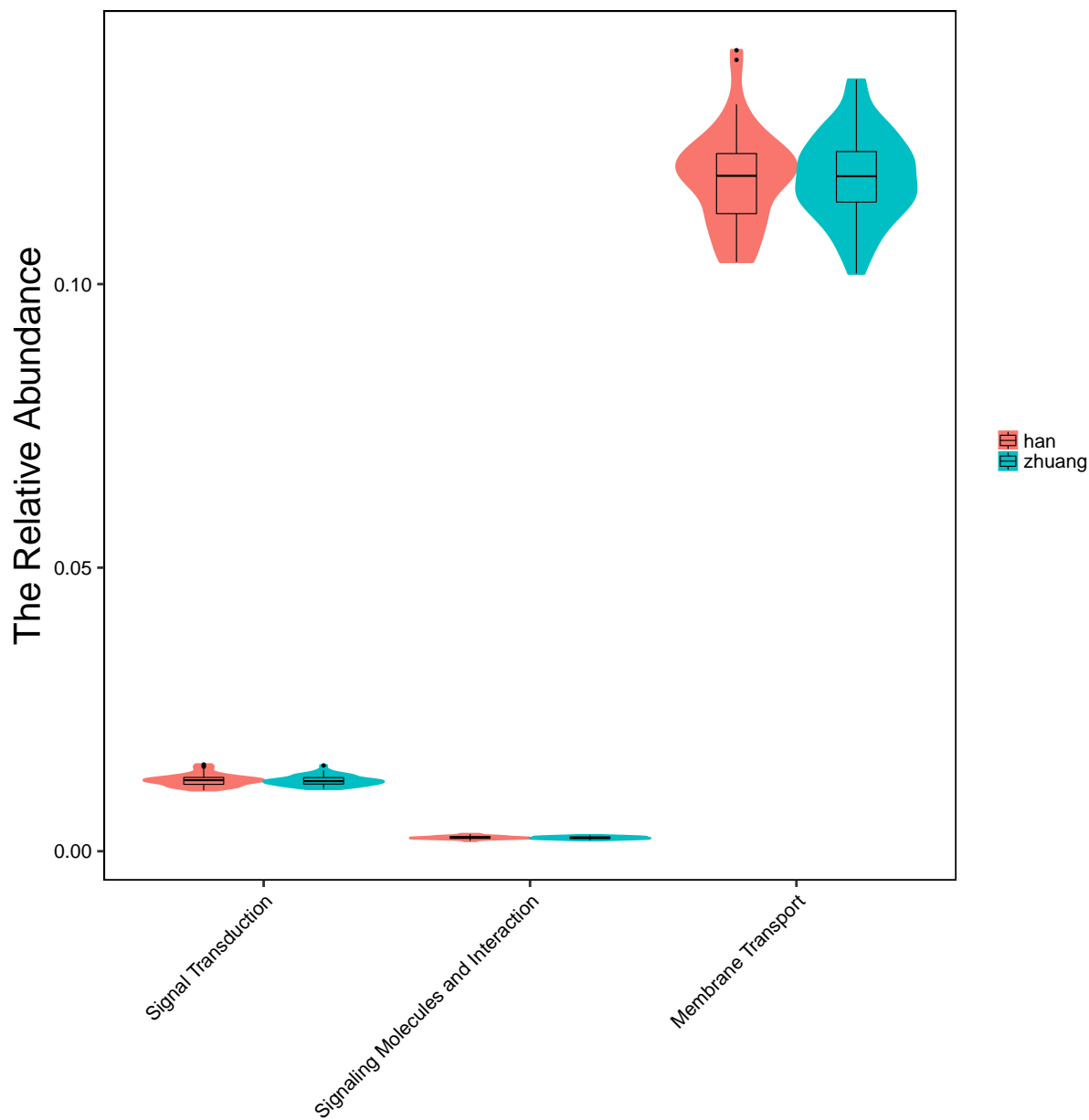

Supplement: Supplementary file 7 [file mmc7.pdf]

# KEGG\_level2: Genetic Information Processing

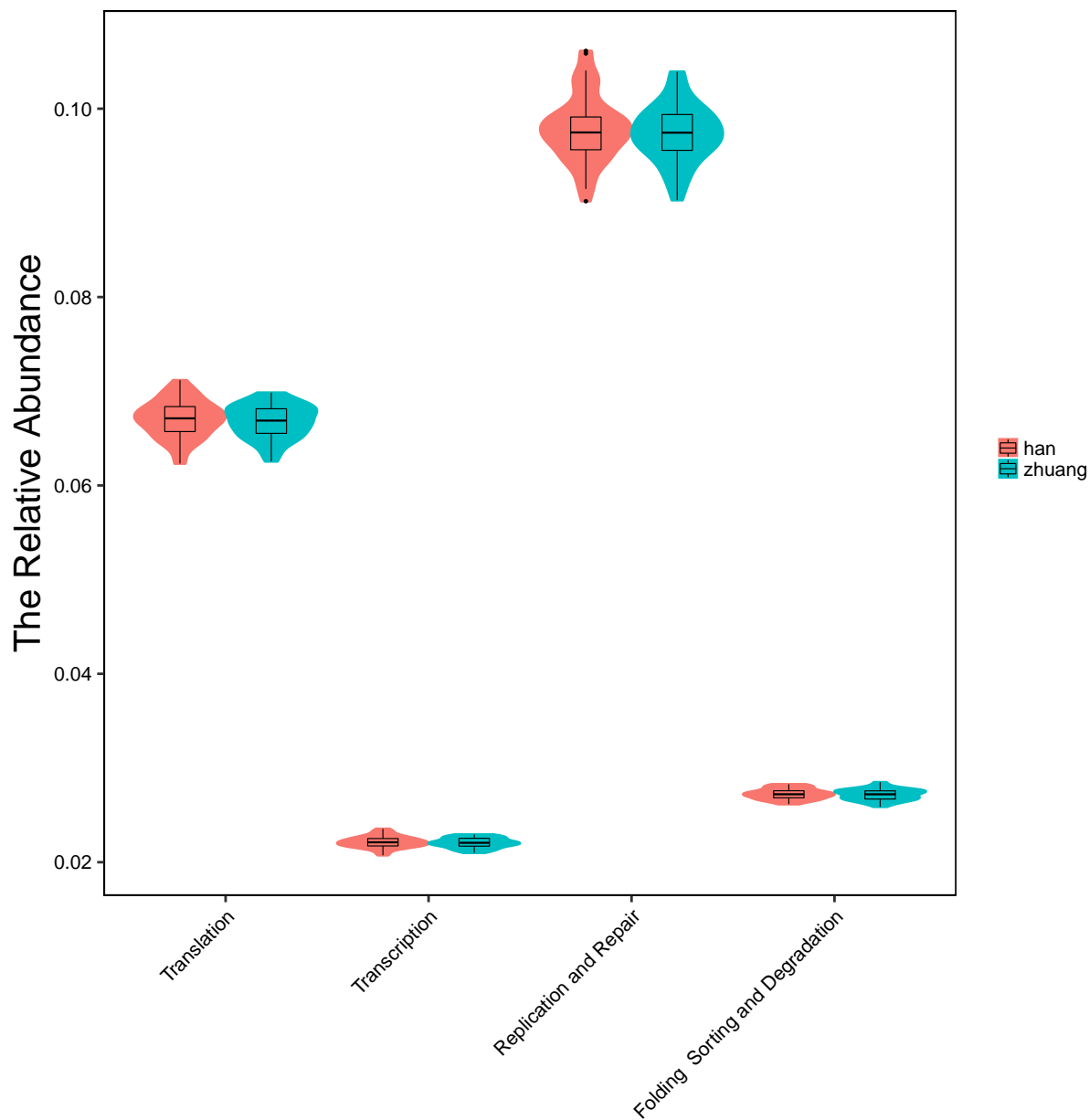

Supplement: Supplementary file 8 [file mmc8.pdf]

# KEGG\_level2: Human Diseases

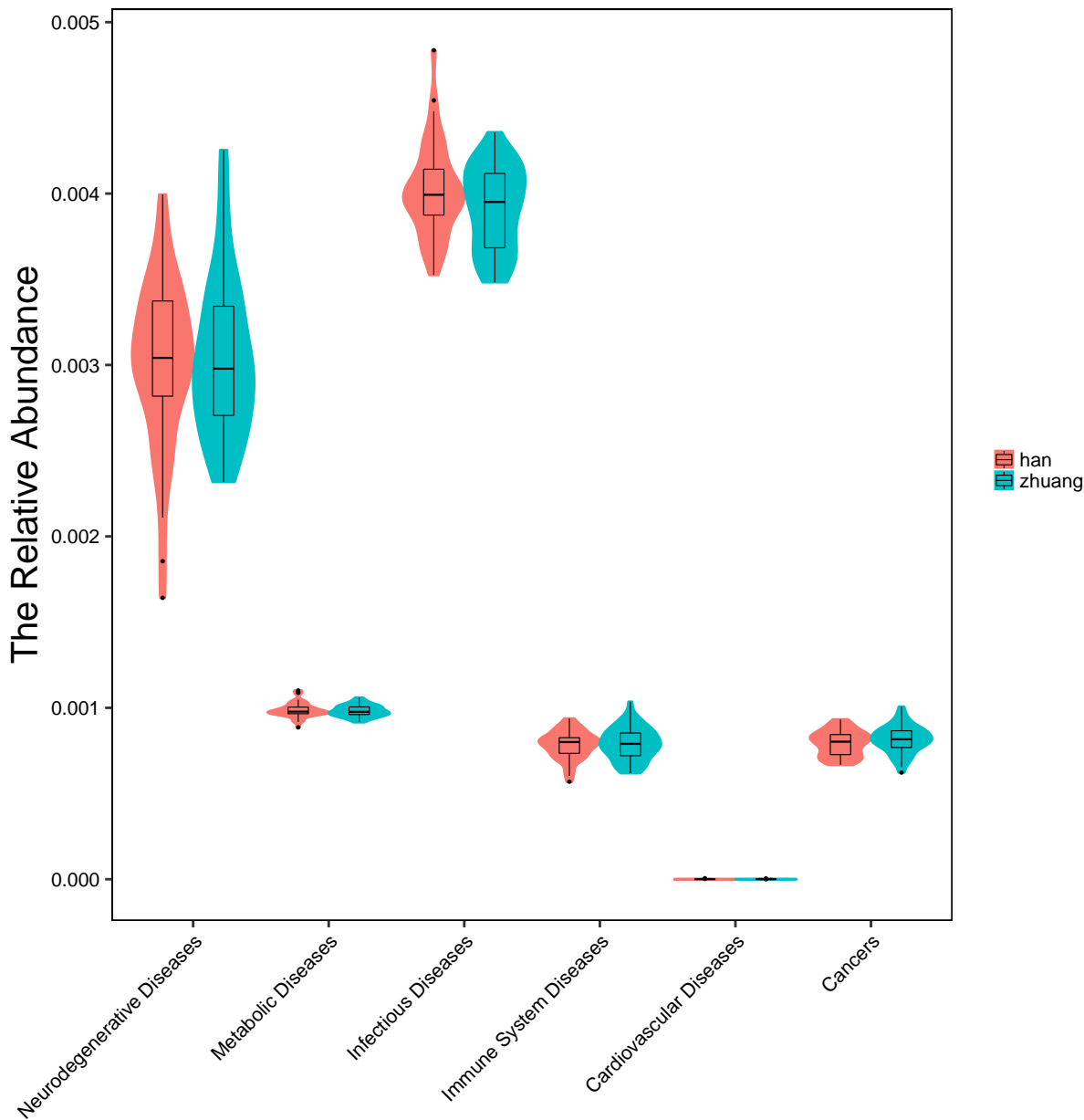

Supplement: Supplementary file 9 [file mmc9.pdf]

# KEGG\_level2: Metabolism

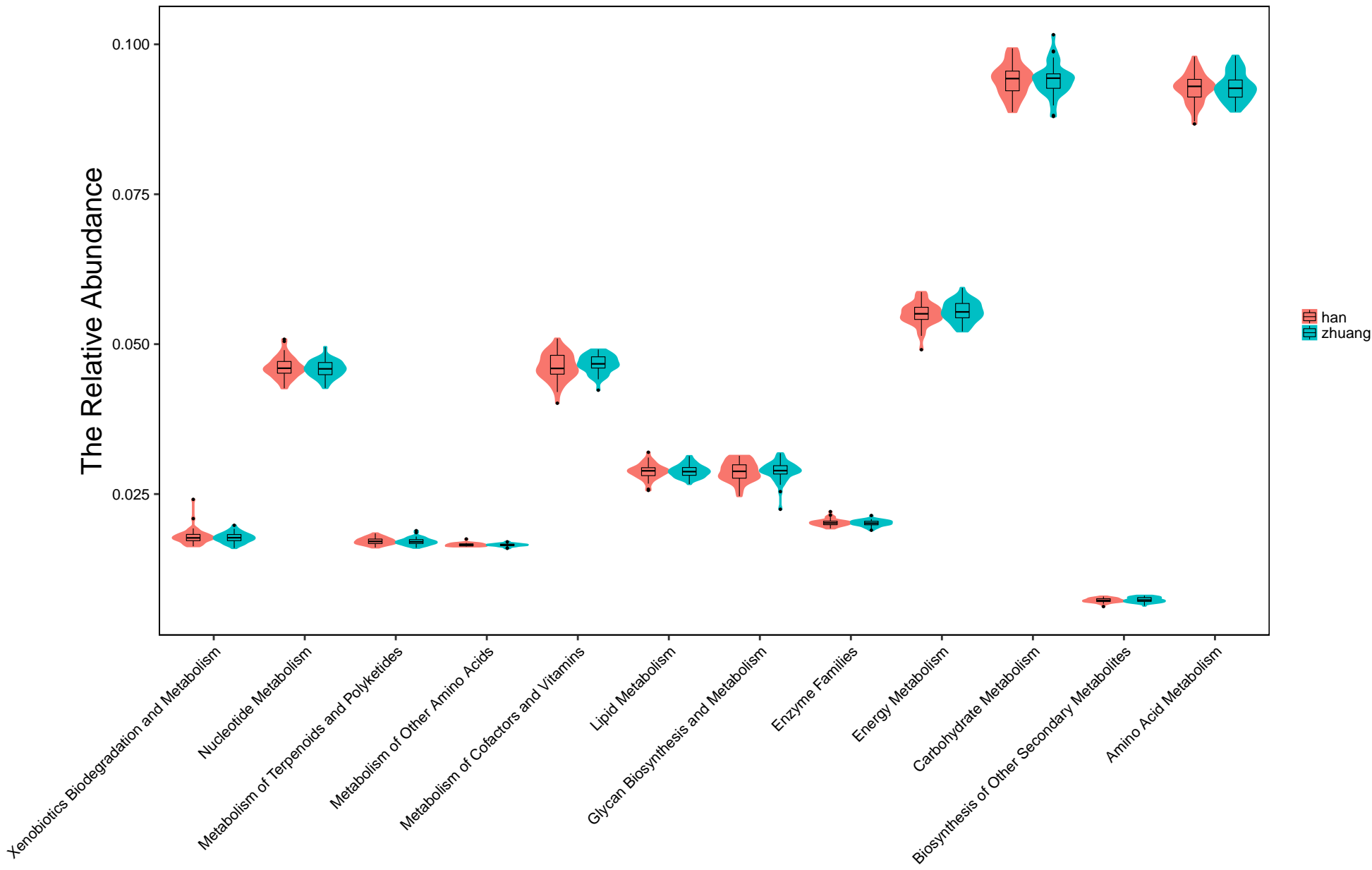

Supplement: Supplementary file 10 [file mmc10.pdf]

# KEGG\_level2: Organismal Systems

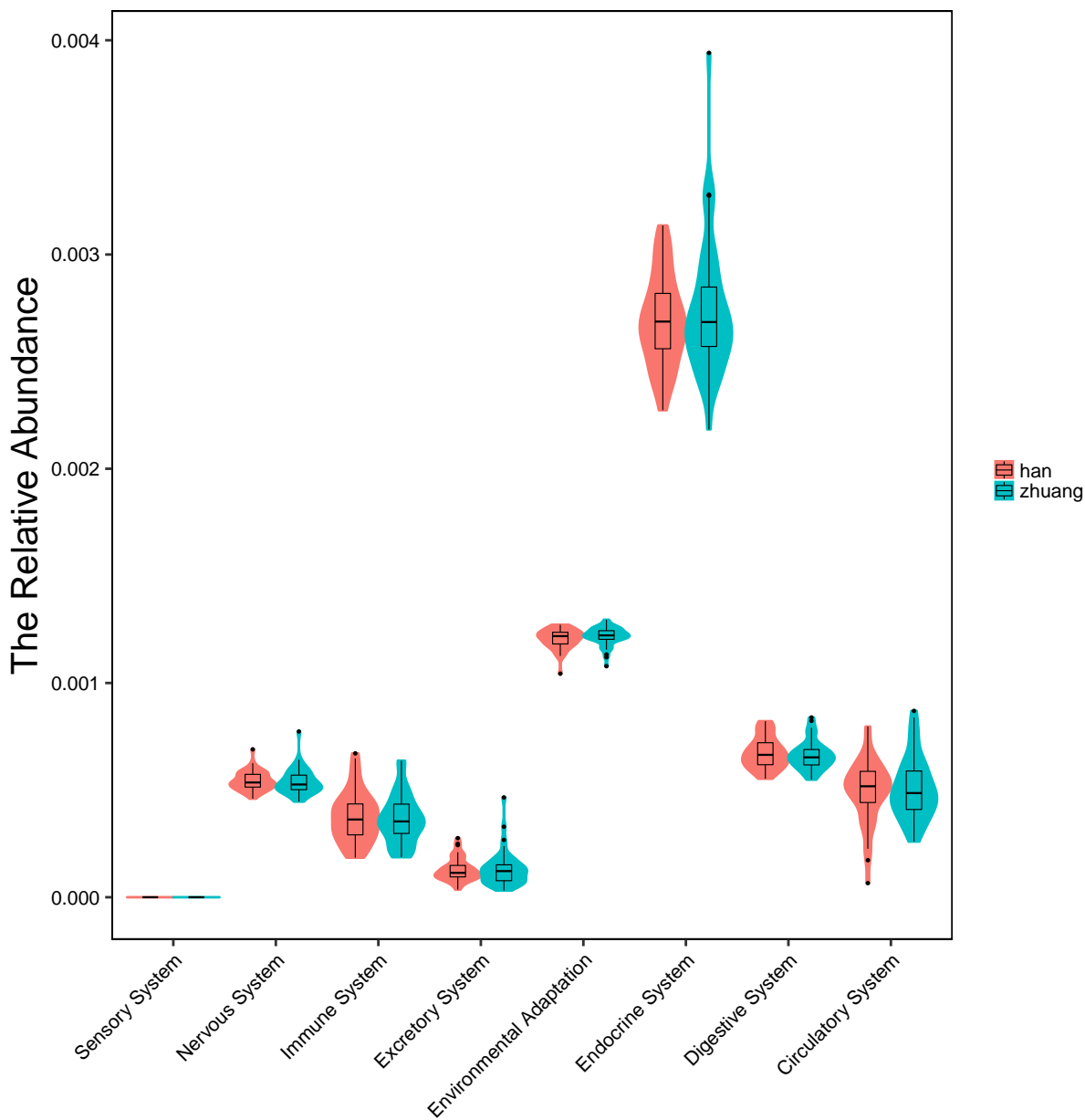

Supplement: Supplementary file 11 [file mmc11.pdf]

# KEGG\_level2: Unclassified

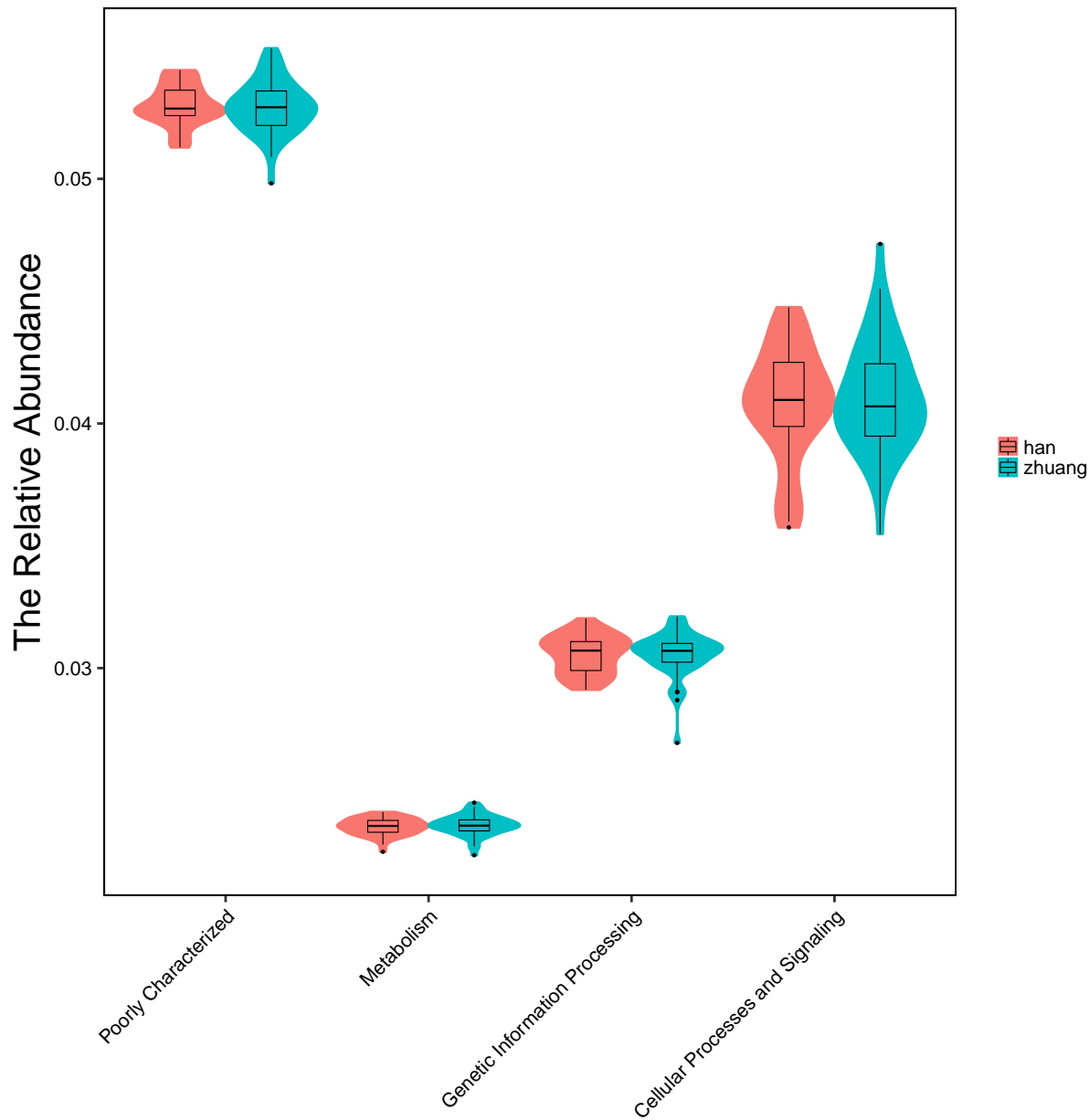

Supplement: Supplementary file 12 [file mmc12.pdf]
